# Spinal Cystic Echinococcosis – A Systematic Analysis and Review of the Literature: Part 2. Treatment, Follow-up and Outcome

**DOI:** 10.1371/journal.pntd.0002458

**Published:** 2013-09-19

**Authors:** Andreas Neumayr, Francesca Tamarozzi, Sam Goblirsch, Johannes Blum, Enrico Brunetti

**Affiliations:** 1 Swiss Tropical and Public Health Institute, Basel, Switzerland; 2 Division of Infectious and Tropical Diseases, University of Pavia, IRCCS S. Matteo Hospital Foundation, WHO Collaborating Centre for Clinical Management of Cystic Echinococcosis, Pavia, Italy; 3 Department of Medicine and Pediatrics, University of Minnesota, Minneapolis, Minnesota, United States of America; Universidad Nacional Autónoma de México, Mexico

## Abstract

Bone involvement in human cystic echinococcosis (CE) is rare, but affects the spine in approximately 50% of cases. Despite significant advances in diagnostic imaging techniques, surgical treatment and introduction of pharmacological therapy, spinal echinococcosis remains associated with a high degree of morbidity, disability and mortality.

We systematically reviewed the published literature of the last five decades to update and summarize the currently existing data on treatment, follow-up and outcome of spinal CE.

## Author Summary

Spinal cystic echinococcosis (CE) is a rare but malignant form of a truly neglected tropical disease. Despite modern diagnostic imaging techniques, the advances in surgery over the last decades and the availability of pharmacological therapy, the clinical management of spinal CE cases remains a challenge for the treating physician. Ten years after the last extensive review on the topic was published by Pamir et al. (Spinal Cord, 2002), we aim to provide clinicians with a systematic review and comprehensive update on spinal CE to help them deal with the often very complex clinical management of these patients. By systematically reviewing all published case reports and case series of spinal CE from 1965 until now, we summarized and analyzed the existing data on treatment, follow-up, and outcome of the disease and discuss the findings in light of the existing data.

## Introduction

*‘Prognosis in vertebral hydatid disease is almost hopeless as regards complete cure. This is due to the impossibility of removing by surgical means all diseased bone, especially if the vertebral bodies are affected, to the multiplicity of extra-osseous cysts, to the certainty of recurrent pressure on the cord…’* (Dew H.R., 1928) [1]

Hydatid disease or cystic echinococcosis (CE), caused by the larval stage of the cestode *Echinococcus granulosus*, is a cosmopolitan parasitic zoonosis. In the accidental human intermediate host, the characteristic cystic lesions are mainly found in the liver (∼70%) and the lungs (∼20%), but virtually any part of the body may be affected, including the bone (∼0.5–4%). The central nervous system (which is involved in ∼3% of all cases) and the vertebral column (which is involved in ≥50% of the ∼0.5–4% of cases affecting the bone) [2]–[5] are particularly vulnerable given the sequelae of their involvement.

‘Spinal CE’ (involvement of the spinal cord, the spine, or both structures) is associated with a high degree of morbidity, disability, and mortality and the prognosis has often been compared to that of malignancies (‘*le cancer blanc*’ [6]), with a case fatality rate reported to exceed 50% within 5 years after onset of symptoms [1], [3], [7], [8].

The treatment of choice is surgical, with removal of the intact cyst(s) being of vital importance. Perforation of the cyst(s) during an operation may lead to systemic dissemination and more critically to local seeding which results in chronic recurrence. Curative surgery remains difficult with bone involvement as infiltration of the bone hampers unruptured and complete resection of the cysts and high recurrence rates plague the long-term outcome [9].

Besides surgery, the only other treatment option for spinal CE is antiparasitic therapy with benzimidazole compounds. Since the introduction of mebendazole (in the 1970s) and albendazole (in the early 1980s), surgery with concomitant and subsequent benzimidazole administration became the widely accepted treatment standard and in cases where surgery is not possible, these drugs remain the only treatment option.

With recurrences presenting many years or even decades after initial treatment of spinal CE, the assessment of treatment outcome remains difficult. Very few retrospective and even fewer prospective long-term follow-up studies are currently available.

We systematically reviewed the published literature of the last five decades for case reports and case series and analyzed and summarized the existing data on treatment, follow-up, and outcome of spinal CE.

## Methods

**Figure 1:**
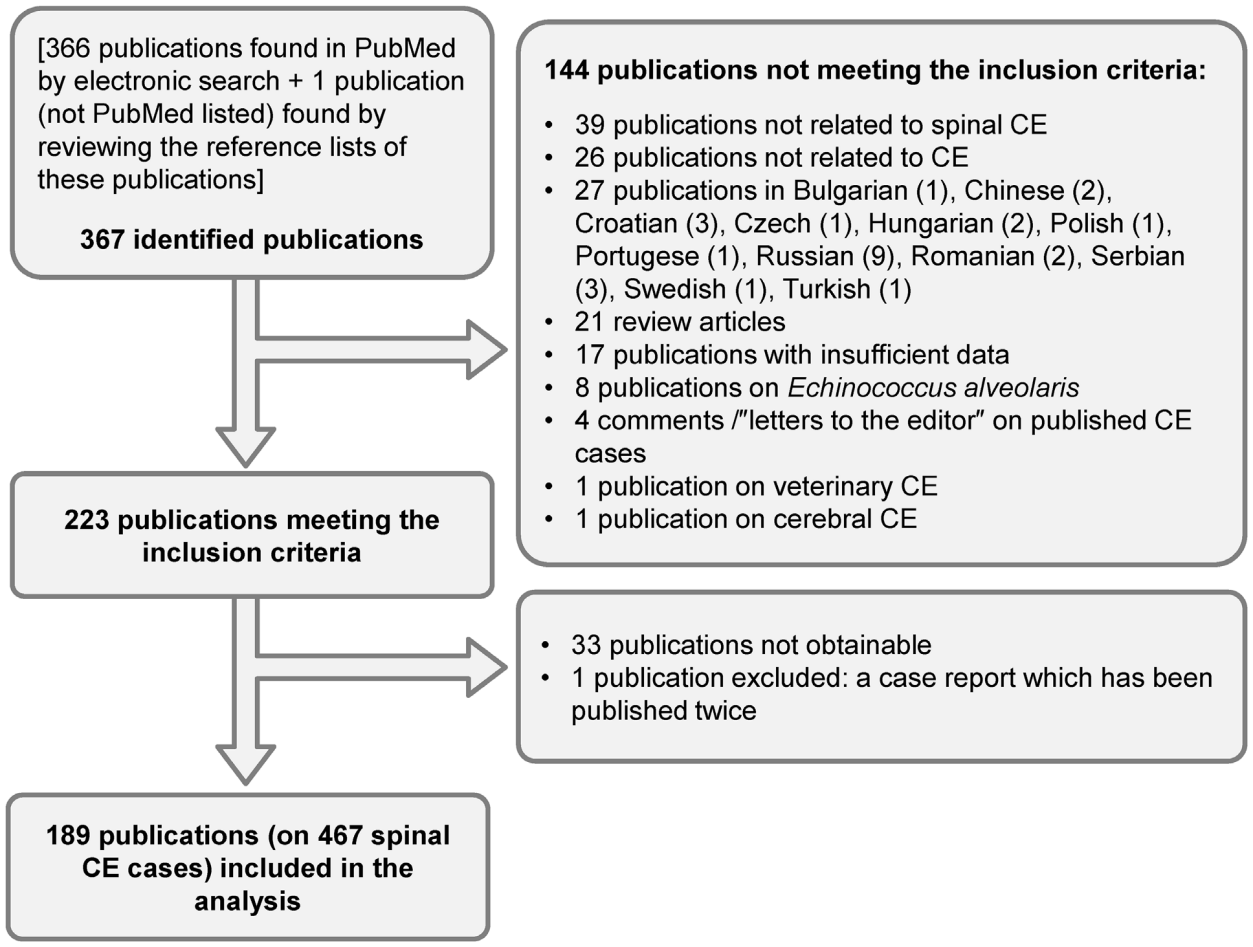
Flow diagram of search and selection of eligible publications.

We performed a PubMed (MEDLINE) search of the literature using the key words ‘spinal echinococcosis’, ‘spinal hydatidosis’, ‘spinal hydatid disease’, ‘spinal echinococcal cyst’, ‘spinal cystic echninococcosis’ and reviewed the obtained references published from 1965 until July 1st 2012 (figure 1; references S 1). The year 1965 was chosen, as it proved difficult to obtain articles before this year.

**Figure 2:**
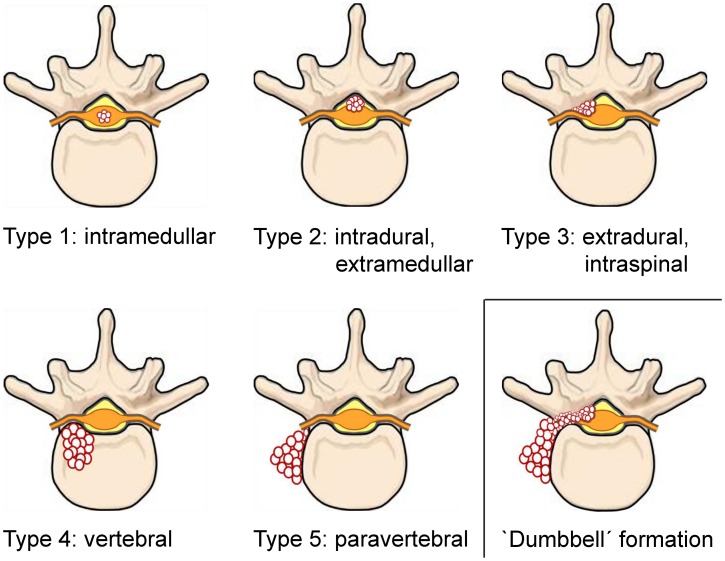
Classification of spinal CE according to the Dew/Braithwaite & Lees classification (type 1–5) and ‘dumbbell’ formation.

All publications on clinical cases and case series of human spinal echinococcosis published in English, French, German, Italian, and Spanish were collected. When the original article was not obtainable but the abstract contained data on anatomy, treatment approach or therapeutic outcome, the publication was included in the analysis. In addition, the reference lists of the collected publications were screened for supplementary (not PubMed listed) case reports on spinal CE eligible for analysis. The collected data included cyst location(s), anatomical structures involved (figure 2), treatment approach(es), surgical technique(s), number of surgical interventions, adjunct medical treatment, clinical outcome, time frame of follow-up, and data on recurrence.

To evaluate the clinical outcome, follow-up time, and recurrence rates of spinal CE with regard to the involved anatomical structures the cases were divided into intradural- extramedullar CE, extradural-extraosseous CE and osseous CE (table 1). The extracted data was entered into Microsoft Excel-files (Version 2002). Data on follow-up periods and recurrence periods was summarized as medians and ranges. Nominal data was summarized as frequencies and percentages.

**Table 1:** Number of operations, clinical outcome, follow-up, and recurrence rates.

Cyst location	No. of cases	No. of operations		Clinical outcome		Follow-up median (range)	Recurrence n (%), Δt median (range)
**Intradural-**	30	1 op.: 28	(93%)	complete recovery:	17 (68%)	10 months	*yes*: 3 (15%), at
**Extramedullar CE**		2 op.: 2	(7%)	partial recovery:	5 (20%)	(2–18)	1.5, 7 & 12 months
**[BL type 2]**				paraparesis:	3 (12%)		*no*: 17 (85%)
				no data:	5	no data: 18	no data: 10
**Extradural extraossous**	70	1 op.: 46	(78%)	complete recovery:	23 (40%)	19 months	*yes*: 12 (27%),
**CE**		2 op.: 10	(17%)	nearly compl. recov.:	7 (12%)	(6–108)	at 22months
**(epidural [BL type 3]-,**		3 op.: 1	(2%)	recovery unspecif.):	4 (7%)		(8–48 m)
**paravertebral [BL type**		6 op.: 1	(2%)	partial recovery:	9 (16%)		*no*: 32 (73%)
**5]-, ‘dumbbell’-, &**		*multiple*: 1	(2%)	paraparesis:	9 (16%)		
**posterior paraspinal**				paraplegia:	4 (7%)		
**muscle cysts)**				fatal postop. complication:	1 (2%)		
		no data:	11	no data:	16	no data: 36	no data: 22
**Vertebral/osseous CE**	169	1 op.: 76	(62%)	complete recovery:	35 (33%)	24 months	*yes*: 50 (48%),
**[BL type 4 & cases**		2 op.: 22	(18%)	nearly compl. recov.:	5 (5%)	(2–144)	at 24 months
**with involvement of**		3 op.: 13	(11%)	recovery (unspecif.):	5 (5%)		(3–156)
**posterior vertebral**		4 op.: 1	(1%)	partial recovery:	32 (30%)		*no*: 53 (52%)
**elements]**		5 op.: 6	(5%)	monoparesis:	1 (1%)		
		6 op.: 2	(1%)	paraparesis:	8 (7%)		
		7 op.: 1	(1%)	paraplegia:	17 (16%)		
		8 op.: 1	(1%)	tetraplegia:	1 (1%)		
				death:	3 (3%)		
		no data:	47	no data:	62	no data: 113	no data: 71

BL: Braithwaite & Lees classification.

## Results

Of the 367 publications identified by electronic search, 189 publications (on 467 cases of spinal CE) were included in the analysis (figure 1).

Data on the treatment approaches, details on the surgical procedures, and data on the use of adjunct medical treatment are shown in tables 2 and 3.

**Table 2:** Treatment approach in 437 spinal CE cases.

Treatment (n = 437)	No. of cases	Surgical approach	N (%)		Notes
Surgery *					
surgical approach	284	posterior only	217	*186*	laminectomy only
specified			(76.4)	*4*	hemilaminectomy only
				*3*	costotransversectomy only
				*24*	laminectomy+posterior stabilization/fusion (incl. pedicle screw-systems, Harrington- & Luque-rods, bone grafting)
		anterior † & posterior	46 (16.2)		laminectomy+various ant. & post. procedures (incl. curettage, vertebrectomy, bone grafting, vertebral body replacement by titanium cage implantation, pedicle screw-systems, Harrington- & Luque-rods, plating)
		anterior † only	21 (7.4)		various procedures (incl. curettage, vertebrectomy, bone grafting, vertebral body replacement by titanium cage implantation, plating)
surgical approach not specified	143				
Medical treatment with albendazole only	8				Belhassen-Garcia 2011 [109], Sudo 2010 [82], Kotil 2010 [87], Tuğcu 2008 [110], Rkain 2007 [111], El Kohen 2003 [83], Kuremu 2002 [84], Poggianti 1997 [112]
PAIR+albendazole	2				Ozdemir 2011 [56], Spektor 1997 [76]

* ± adjunct medical treatment with benzimidazoles (see table 3).

† *anterior*: transthoracic, transabdominal, retroperitoneal, and transoral.

**Table 3:** Surgery and adjunct benzimidazole treatment in 420 spinal CE cases.

Surgery & adjunct benzimidazole treatment*	No. of cases	Notes
Adjunct medical treatment mentioned:		
Surgery+benzimidazole treatment	151	median time of treatment [n = 106]:
		6 months (3 weeks - indefinite) dosages:
		albendazole: 10–15 mg/kg/d
		mebendazole: 40–60 mg/kg/d
Surgery+benzimidazole+praziquantel	12	*Lam 1997* (1 case) [59]; *Keller 1997* (1 case) [35]; *El-On 2003* (1 case) [48]; *Hamdan 2012* (9 cases) [38]
Surgery without adjunct medical treatment	2	*Pandey 1997* (unruptured resection of a single cyst from the sacral spinal canal) [89]; *Hamdan 2000* (total excision of a thoracic dumbbell hydatid cyst) [88]
No adjunct medical treatment mentioned	174	
Published before the availability of benzimidazoles	81	

* albendazole or mebendazole.

## Discussion

### Surgical treatment

Almost two centuries after Reydellet performed the first surgical intervention on a patient with spinal CE (1819), surgery remains the treatment of choice [7]. Depending on location and extent of disease, the two primary tasks in surgery of spinal CE are *decompression* of a compromised spinal cord and *stabilisation* of a compromised spinal column. Table 2 summarizes the frequencies of the different surgical approaches we found in the reviewed literature and highlights the significance of posterior decompression by laminectomy, which is performed in over 90% of all cases.

#### Intradural CE

Patients with cysts confined to the intradural space typically present with acute to subacute compression of the spinal cord and require urgent decompression. Intradural cysts are approached posteriorly via laminectomy followed by durotomy. The complete resection of the cysts without rupture is considered curative and feasible in most cases, even in the presence of adhesions to surrounding tissue [10]–[14]. In some cases the excision of dura tissue together with the adherent cyst might be necessary [12]. However, the cyst walls are very thin and fragile and rupture might even occur at removal of free floating cysts with only minimal arachnoidal adhesions [15]. In extensive intradural disease, complete resection might be impossible [16]. The rapid development of neurological symptoms leading to early diagnostic work-up and surgery is considered to be responsible for the favorable outcome of most patients [17]. The favorable outcome might also be attributed to the fact that intradural CE is often limited to a single cyst. In a series of 72 spinal CE cases the recurrence rate (RR) of intradural CE (n = 10) was 0% and statistically significant lower than the RR of extradural-intraspinal (n = 17; RR = 6%), paraspinal (n = 3; RR = 33%), or vertebral CE (n = 41; RR = 32%) [18]. The subgroup analysis of our collected data (3 recurrences among 20 cases [15%]) confirms the favorable outcome of intradural CE (table 1). Of note, in two of the three cases an explanation for recurrence was reported: rupture of cysts at initial surgery in one case [15] and incomplete resection of the cyst in the other [19].

#### Extradural-extraosseous CE

The spectrum of extradural-extraosseous CE ranges from single epidural or paravertebral cysts to large dumbbell formations and cases with extensive soft tissue infiltration. As all epidural cysts and the majority of dumbbell cysts present with spinal cord compression, decompression by laminectomy is almost always part of the surgical management. To reach paravertebral lesions, an anterior approach is usually needed. In a case where complete excision of an intact dumbbell cyst was not feasible, intraoperative incision and aspiration with adjacent resection of the reachable cyst wall has been described (without recurrence over 18 months follow-up) [20]. One case has been published where intra- and parasacral cysts were successfully removed by rigid endoscopy [21]. The subgroup analysis of our collected data yields an overall recurrence rate of 27% (table 1) for extradural-extraosseous CE, which is within the reported range of 6% (extradural-intraspinal CE) - 33% (paraspinal CE) [18]. The two main factors determining recurrence (*complete resection* and *unruptured resection*) might explain the higher recurrence rate of extradural-extraosseous CE: the higher frequency of multiple cysts and the higher rate of intraoperative cyst rupture. In a large review on CE of the central nervous system, Altinoers and colleagues found an intraoperative rupture rate of 16.9% for intracranial lesions and 44.4% for intraspinal cysts, which they attributed to the discrepancy between the rather narrow space in spinal CE with close bony boundaries and the often multiple spinal lesions [22]. Islekel and colleagues commented that even laminectomy almost always leads to rupture of extradural cysts [13].

#### Vertebral/osseous CE

In spinal CE involving bone, the infiltrative nature of the microvesicular lesions does not allow for unruptured resection of the cysts and therefore spillage of their contents is inevitable [13], [17], [18], [23]. In addition, the extent of disease may prohibit complete resection of the affected bone and progressive vertebral destruction may compromise stability of the spinal column [3].

If possible, radical surgery is advocated and although often not precluding disease progression, it has been shown to prolong survival in selected cases [24]. The location and the extent of the lesion(s) determine the surgical approach and the necessary surgical procedure (table 2). After resection of affected bone, a wide spectrum of techniques has been employed to stabilize the vertebral column (including bone grafts [17], [25]–[43], dorsal stabilisation by pedicle screw systems [9], [26], [27], [37], [44]–[48], Harrington- [9], [27], [36], [39], [49]–[51] and Luque- [9], [45], [50], [51] rods, vertebral body replacement by titanium cage implantation [44], [52], [53], plating [25], [35], [52], [54], bone cement [26], [55], [56]). However, in the absence of evidence no recommendations regarding the use of a specific technique can be given besides possibly bone grafting. Considering that osseous grafts might be invaded by persisting or recurrent disease, the use of acrylic cement for osteosynthesis has been discussed [27], and in a case series of 10 patients with bone CE (including 2 cases with spinal CE) Yildiz and colleagues reported that the use of polymethylmethacrylate (PMMA) cement lowered recurrence rate and prolonged the interval between recurrences [55]. Regarding the surgical approach, two rare cases of spinal CE affecting the craniocervical junction might be worth mentioning for their surgical approach via an enoral route [33], [43]. Among the reviewed cases with vertebral bone involvement we found an overall recurrence rate of 48%, but the stage of disease among the cases varied widely and data on follow-up was mostly scarce (table 1). The available literature states a recurrence rate of 30–100% [18], [24], [28], [38], [44], [57], [58] and in advanced cases interventions might generally be considered palliative with recurrence inevitable [3], [38]. However, remarkable cases with extensive vertebral CE, multiple palliative operations and prolonged survival over many years and even decades have been described [59]–[61].

A major limitation of the available data on recurrence of spinal CE is the overall short follow-up period throughout the literature (table 2) and only a few case series shed light on the long-term prospects [3], [9], [24], [38], [55], [57]. To obtain solid data on the long-term outcome of the various forms of spinal CE, larger numbers of cases and follow-up over many years, possibly even decades, would be required. With follow-up periods generally not much longer or even equal (table 1) to the observed recurrence intervals, the current existing data probably presents an optimistic lower limit of recurrence rates in spinal CE.

Various scolicidal agents are routinely used in surgery and percutaneous treatment of visceral CE to sterilize the cysts at intervention, with 95% ethanol and 20% hypertonic saline being the most frequently used solutions [62]. *In vitro* studies have demonstrated a dose- and time- dependent effect: the higher the concentration and the longer the exposure time, the greater and faster the scolicidal effect [63], [64]. As rupture of cysts at surgery of spinal CE is a central problem, most surgeons advocate the use of scolicidal agents to irrigate the operative field, although the efficacy of this measure remains unproven and potentially deleterious effects on neural tissue have to be considered [7], [18], [65]. Various agents have been deployed: 3%–7.2% hypertonic saline, povidone iodine, ethacrine lactate (rivanol), hydrogen peroxide, silver nitrate, cetrimide, formalin [9], [18], . In a case series of 66 spinal CE cases no significant difference in recurrence rate was observed between the intraoperative use of 3% saline-, 10% formaldehyde- or 0.5% silver nitrate/povidone iodine-solution [18]. However, in light of the above mentioned *in vitro* studies, the use of hypertonic saline with concentrations lower than 20% appears to be questionable. The use of 20% hypertonic saline in spinal CE has been reported and appears to be safe even for intradural irrigation [13], [26], [58], [72]. Ten per cent formaline solution is considered safe in cranial CE, but possibly dangerous in spinal CE: a fatal case related to toxic myelitis following dural tear has been published [13]. A case of coma and hemolysis following epidural irrigation with cetrimide has been reported [73]. Even though scolicidal agents are not active against intact microscopic daughter cysts (which demand chemotherapy), they are effective against spilled protoscolices from ruptured cysts and therefore considered a valuable adjunct to radical surgery and chemotherapy [4].

Besides leading to disease recurrence, the spillage of cyst content during surgery may provoke various hypersensitivity reactions, ranging from pruritus, urticaria, rash, edema, bronchospasm, and gastrointestinal symptoms to hypotension and fatal anaphylactic shock [74]. Although frequently being quoted, data on the incidence of anaphylactic reactions during surgery of CE (including spinal CE) remains scarce. Among the reviewed cases we found only two reports on severe anaphylactic reactions during surgery: a case of cardiovascular collapse and bronchospasm [9] and a case where resection of lesions adhering to the abdominal aorta led to arterial dissemination of daughter cysts and fatal anaphylaxis [38]. Even though anaphylactic reactions are rarely reported, it has to be considered, that non-severe anaphylactic reactions may remain undetected under general anaesthesia [75]. Some authors have advocated the use of glucocorticosteroids to prevent anaphylactic shock during surgery [26], [76]. It has been observed that the risk for anaphylactic shock appears to rise with the number of surgical operations and therefore some authors have used intradermally injected hydatid echinococcal antigen for ‘desensitization’ [61], [67]. The same approach has also been tried as ‘biological therapy’ [77], [78]. However, data remained disputable and both approaches were abandoned when medical treatment with mebendazole became available.

### Medical treatment

Therapy with benzimidazole derivatives has shown to be effective in visceral CE [79] although a recent systematic review concluded that treatment response depends on cyst stage and size, and that efficacy of medical treatment may have been overstated in the past [80].

Data on the efficacy of benzimidazoles in osseous CE is scarce and the contribution of these drugs to cure or even prevent recurrence in vertebral CE is frequently debated [38], [57]. Todorov and colleagues investigated factors influencing the efficacy of benzimidazoles and found, besides larger cyst size and the presence of daughter cysts, especially bone involvement to be unfavorable [81].

However, various publications have reported favorable results attributed to medical treatment: several authors have used albendazole in patients with inoperable spinal CE (table 3) and some reported favorable results even in cases with extensive disease [59], [82]–[84]. In a study of 40 patients with inoperable vertebral hydatitosis El-Mufti and colleagues reported a cure rate of 53% with albendazole treatment at a minimal follow-up time of two years [85]. From long-term follow-up of surgical spinal CE cases Islekel and colleagues concluded that adjunct pharmacotherapy appears to retard recurrence [13]. The successful medical treatment of a residual cyst (post surgery) with a one year course of albendazole has been reported (follow-up time at publication: one year) [86]. Kotil and colleagues reported successful albendazole treatment of a solitary extradural lumbar cyst in a patient presenting with mild neurological symptoms and concluded that medical treatment might be suitable for patients with solitary lesions in the absence of indications for immediate surgery (no neurological deficit or spinal instability) [87].

Among the reviewed cases, adjunct pharmacotherapy was used in 98.8% of all cases (table 3). In the only 2 cases where deliberately no adjunct medical treatment was given, the authors reported complete resection without spillage of the cysts [88], [89].

In the absence of pharmacokinetic data on benzimidazoles in bone or spinal soft tissue, most authors use the dosages recommended by the World Health Organisation (WHO) for visceral CE (mebendazole 40–50 mg/kg/day; albendazole 10–15 mg/kg/day [90]), with albendazole being preferred over mebendazole due to the better pharmacokinetic properties and superior efficacy [91], [92]. Considering the high to inevitable risk of cyst rupture, perioperative treatment with albendazole should be initiated ≥4 hours before surgery to achieve scolicidal blood levels [93]. The addition of praziquantel to albendazole is sometimes advocated due to the protoscolicidal effect of praziquantel. However, data on the efficacy of this approach is currently missing [94]. The optimal duration of medical treatment has not been determined, neither for visceral CE nor for osseous CE or the various forms of spinal CE. Among the reviewed cases most authors preferred 6 months of treatment (table 3). In some cases with extensive disease even indefinite medical treatment has been reported [53].

Due to the observed high inter-individual variablity of maximal albendazole sulphoxide levels and taking into account drug-interactions, it has been proposed to increase albendazole bioavailability (and blood levels of the active metabolite albendazole sulphoxide) by coadministration of cimetidine. However, data on interactions of cimetidine with intestinal absorption and hepatic metabolization of albendazole remains conflicting and the issue controversial [95], [96].

Medical treatment with albendazole might not be able to cure or prevent recurrence of spinal CE, but it remains the only treatment option for inoperable patients and the use of drug therapy in conjunction to surgery is generally considered useful to decrease the risk of recurrence, delay recurrence and prevent seeding from intraoperative cyst spillage.

Besides benzimidazoles, few other medical treatment options have been tried in osseous CE and their role remains to be defined:

Among the reviewed cases, we found four publications where albendazole plus praziquantel combination therapy was used (table 3) [35], [38], [48], [59]. As these were either case reports or case series without a control group, the efficacy of this combination remains to be defined. Like cimetidine, praziquantel enhances the bioavailability of albendazole [97], [98].

Three publications on five cases of osseous CE and adjunct nitazoxanide treatment have been published [99]–[101]. As these were case reports, the efficacy of adjunct nitazoxanide treatment remains to be defined.

### Percutaneous treatment

We found two cases where CT-guided percutaneous treatment using the PAIR approach (Puncture of the cyst, Aspiration of the cyst fluid, Injection of a scolicidal agent, and Re-aspiration of the cyst content) has been reported (table 2).

Spektor and colleagues reported successful percutaneous treatment in a patient with a cervical vertebral hydatid cyst (C2-3) extending into the spinal canal and causing severe spinal cord compression. The intervention led to rapid decompression and clinical improvement. No adverse events were observed and follow-up over one year showed favorable imaging results with complete resolution of clinical symptoms. The authors concluded that percutaneous treatment might be particularly beneficial in cases with extensive disease where radical removal is impossible [76].

Ozdemir and colleagues reported the use of PAIR in a patient who had undergone complex surgery for thoracic spinal CE before (partial corpectomy, posterior fusion with transpedicular screws, bone cement) and presented with severe paraparesis due to recurrent disease. Two vertebral cysts extending into the spinal canal and causing severe spinal cord compression were successfully treated by bilateral transpedicular interventions. No adverse events were observed and follow-up over six months showed favorable imaging results with complete resolution of clinical symptoms. The authors concluded that percutaneous treatment should be considered in cases where prior surgical interventions aggravate re-operation [56].

### Radiotherapy

In 1965 Fitzpatrick reported the use of radiotherapy in a case with extensive spinal CE and noted that no apparent benefit of this treatment accrued [61]. Sporadically, the use of radiotherapy as an adjunct to surgery and medical treatment has been reported in cerebral and osseous CE, but data regarding the efficacy of this approach is pending [22], [102], [103]. Experimental studies evaluated the use of radiotherapy in rodent models and depicted a dose depending effect on scolex viability in osseus CE [104] and inhibition of *E. multilocularis* larval growth [105]–[107]. Recently a case report describing the successful use of radiotherapy in a patient with sternal *E. multilocularis* infection with failed multiple surgical procedures and medical treatment has been published [108].

### Conclusion

Despite significant advances in diagnostic imaging techniques and surgical treatment, spinal echinococcosis remains associated with a high degree of morbidity, disability and mortality.

Surgery is the treatment of choice with *decompression* of a compromised spinal cord and *stabilization* of a compromised spinal column being the two primary tasks.

The long-term outcome depends on complete resection of all parasitic lesions, which is frequently hampered by the infiltrative nature of the disease.

Recurrence rates of spinal CE are high, especially in cases with vertebral bone involvement.

The currently existing data on follow-up of spinal CE cases is largely insufficient to judge the long-term outcome of surgery.

Besides surgery, the intraoperative use of scolicidals and the pre-, peri-, and postoperative use of albendazole is currently considered the treatment standard of spinal CE.

The role of combination drug therapy, percutaneous treatment and radiotherapy in spinal CE remains to be defined.

## Supporting Information

Checklist S1 PRISMA checklist. 27-item checklist for systematic reviews.
(DOC)

Diagram S1 PRISMA flow diagram. Flow of information through the different phases of the systematic review.
(DOC)

References S1 Reference list of included and excluded publications.
(DOC)
