# Supplementary material for: Spinal Cystic Echinococcosis – A Systematic Analysis and Review of the Literature: Part 2. Treatment, Follow-up and Outcome
Source: PLoS Negl Trop Dis. 2013 Sep 19;7(9):e2458. doi: 10.1371/journal.pntd.0002458 (PMC3777903; doi:10.1371/journal.pntd.0002458)
Supplement: Diagram S1 — PRISMA flow diagram. Flow of information through the different phases of the systematic review. (DOC) [file pntd.0002458.s002.doc]

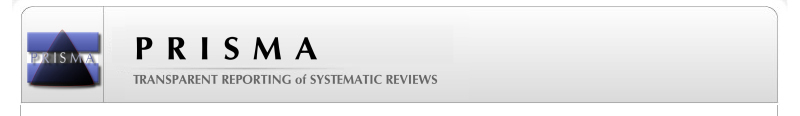
**PRISMA 2009 Flow Diagram**

**Screening**

**Included**

**Eligibility**

**Identification**

Records identified through database searching
(n = 366)

Additional records identified through other sources
(n = 1)

Records after duplicates removed
(n = 367)

Records screened
(n = 367)

Records excluded
(n = 144)

Full-text articles assessed for eligibility
(n = 223)

Full-text articles excluded, with reasons
(n = 34)

Studies included in qualitative synthesis
(n = 189)

Studies included in quantitative synthesis (meta-analysis)
(n = 189)
