# Supplementary material for: Spinal Cystic Echinococcosis – A Systematic Analysis and Review of the Literature: Part 2. Treatment, Follow-up and Outcome
Source: PLoS Negl Trop Dis. 2013 Sep 19;7(9):e2458. doi: 10.1371/journal.pntd.0002458 (PMC3777903; doi:10.1371/journal.pntd.0002458)
Supplement: References S1 — Reference list of included and excluded publications. (DOC) [file pntd.0002458.s003.doc]

**Reference list of included and excluded publications**

**References of included publications**

1. Abdelmoula Cheikhrouhou L, Amira C, Chaabouni L, Ben Hadj Yahia C, Montacer Kchir M, et al. (2005) [Vertebral hydatidosis: medical imaging and management. A case report]. Bull Soc Pathol Exot 98: 114-117.

2. Acikgoz B, Sungur C, Ozgen T, Camurdanoglu M, Berker M (1996) Endoscopic evacuation of sacral hydatid cysts: case report. Spinal Cord 34: 361-364.

3. Adilay U, Tugcu B, Gunes M, Gunaldi O, Gunal M, et al. (2007) Cauda equina syndrome caused by primary lumbosacral and pelvic hydatid cyst: a case report. Minim Invasive Neurosurg 50: 292-295.

4. Akhan O, Dincer A, Saatci I, Gulekon N, Besim A (1991) Spinal intradural hydatid cyst in a child. Br J Radiol 64: 465-466.

5. Aniba K, Khoumiri R, Aitbenali S, Maksi B, Oussehal A, et al. (2004) [Echinococcosis located at spinal canal and vertebrae]. Arch Pediatr 11: 1510-1511.

6. Arazl M, Memlk R, Kapicioglu MI (1998) Answer please. Hydatid disease of the spine. Orthopedics 21: 912, 909-910.

7. Arif SH, Zaheer S (2009) Intradural extramedullary primary hydatid cyst of the spine in a child: a very rare presentation. Eur Spine J 18 Suppl 2: 179-182.

8. Awasthy N, Chand K (2005) Primary hydatid disease of the spine: an unusual case. Br J Neurosurg 19: 425-427.

9. Basak M, Ozel A, Yildirim O, Erturk M (2002) Relapsing hydatid disease involving the vertebral body and paravertebral soft tissues. Acta Radiol 43: 192-193.

10. Baurand C, Daniel F, Guillermain P, Tournigand T, Gascard E (1970) [Spinal cord compression by an intra-dural hydatid cyst (apropos of a case)]. Mars Med 107: 921-922.

11. Bavbek M, Inci S, Tahta K, Bertan V (1992) Primary multiple spinal extradural hydatid cysts. Case report and review of the literature [corrected]. Paraplegia 30: 517-519.

12. Baykaner MK, Dogulu F, Ozturk G, Edali N, Tali T (2000) A viable residual spinal hydatid cyst cured with albendazole. Case report. J Neurosurg 93: 142-144.

13. Baysefer A, Gonul E, Canakci Z, Erdogan E, Aydogan N, et al. (1996) Hydatid disease of the spine. Spinal Cord 34: 297-300.

14. Belhassen-Garcia M, Carpio-Perez A, Blanco JF, Velasco-Tirado V, Pardo-Lledias J (2011) Recurrent spinal echinococcosis. Int J Infect Dis 15: e435-436.

15. Berk C, Ciftci E, Erdogan A (1998) MRI in primary intraspinal extradural hydatid disease: case report. Neuroradiology 40: 390-392.

16. Bettaieb A, Khaldi M, Ben Rhouma T, Touibi S (1978) [Spinal echinococcosis; clinical study of 32 cases (author's transl)]. Neurochirurgie 24: 205-210.

17. Bhatoe HS, Bakshi SS, Swamy GL (2000) Trichinoechinococcosis T-5. Case report. J Neurosurg 92: 225-228.

18. Bhojraj SY, Shetty NR (1999) Primary hydatid disease of the spine: an unusual cause of progressive paraplegia. Case report and review of the literature. J Neurosurg 91: 216-218.

19. Booz MK (1972) The management of hydatid disease of bone and joint. J Bone Joint Surg Br 54: 698-709.

20. Bouden A, Khaled S, Annabi H, Mbarek M, Kraiem NH, et al. (2006) [Unusual localization of spinal hydatid cyst]. Tunis Med 84: 201-204.

21. Bouklata S, El Mahi M, Karmouni W, El Hassani MR, Chakir N, et al. (2000) [Isolated dorsal extradural hydatid cyst. A case report]. J Neuroradiol 27: 285-286.

22. Bozbuga M, Celikoglu E, Boran BO (2005) Hydatid cyst of the craniocervical junction: case report. Neurosurgery 57: E193; discussion E193.

23. Braithwaite PA, Lees RF (1981) Vertebral hydatid disease: radiological assessment. Radiology 140: 763-766.

24. Bron JL, van Kemenade FJ, Verhoof OJ, Wuisman PI (2007) Long term follow-up of a patient with disseminated spinal hydatidosis. Acta Orthop Belg 73: 678-682.

25. Carta F, Perria C, Davini V (1974) Vertebral echinococcosis. J Neurosurg Sci 18: 228-232.

26. Celik C, Sasmaz MF, Oktay F, Ucan H, Kaptanoglu E (2010) Paraplegia associated with spinal hydatid cyst: a case report. Spine (Phila Pa 1976) 35: E356-358.

27. Chakir N, Akhaddar A, El Quessar A, El Ouahabi A, El Hassani MR, et al. (2002) [Primary intradural extramedullary hydatidosis. Case report and review of the literature]. J Neuroradiol 29: 177-182.

28. Charles RW, Govender S, Naidoo KS (1988) Echinococcal infection of the spine with neural involvement. Spine (Phila Pa 1976) 13: 47-49.

29. Charrada-Ben-Farhat L, Said W, Bouslama K, Oueslati S, Dridi L, et al. (2006) [Vertebral and spinal cord hydatidosis: contribution of imaging]. Med Mal Infect 36: 58-61.

30. Chat L, Akjouj M, Chellaoui M, Gueddari F, Alami D, et al. (2000) [Spinal intradural hydatid cyst. A case report]. J Radiol 81: 535-537.

31. Chikhaoui N, Adil A, Kadiri R (1993) [Radiological aspects of vertebro-medullary hydatid cysts. Apropos of 12 cases]. J Radiol 74: 621-628.

32. Cogan A, Ilharreborde B, Lenoir T, Hoffmann E, Dauzac C, et al. (2011) Hydatid disease drug therapy primarily to reconstruction of a multilevel thoracolumbar vertebral lesion. Orthop Traumatol Surg Res 97: 766-769.

33. De Simone M, Mazzuca L, Tenna M (1988) [A white tumor: a fatal case of multiple vertebral hydatidosis]. Riv Eur Sci Med Farmacol 10: 487-491.

34. Doganay S, Kantarci M (2009) Role of conventional and diffusion-weighted magnetic resonance imaging of spinal treatment protocol for hydatid disease. J Spinal Cord Med 32: 574-577.

35. El Kohen A, Benjelloun A, El Quessar A, Derraz S, Lazrak A, et al. (2003) Multiple hydatid cysts of the neck, the nasopharynx and the skull base revealing cervical vertebral hydatid disease. Int J Pediatr Otorhinolaryngol 67: 655-662.

36. El Quessar A, Jroundi L, Tizniti S, Cisse M, Chakir N, et al. (2001) [CT and MRI features of spinal hydatidosis. A report of 8 cases]. J Radiol 82: 917-921.

37. El-On J, Ben-Noun L, Galitza Z, Ohana N (2003) Case report: clinical and serological evaluation of echinococcosis of the spine. Trans R Soc Trop Med Hyg 97: 567-569.

38. Eloqayli H, Matalka I, Daoud S (2010) Primary spinal extradural hydatid cyst in a 4-year-old child. Br J Neurosurg 24: 602-603.

39. Emara KM, Abd Elhameed DM (2007) Hydatid disease of the lumbar spine: combined surgical and medical treatment--a case report. Am J Orthop (Belle Mead NJ) 36: E12-14.

40. Erayman I, Kalkan E, Erdi F, Kerimoglu U, Esen H (2011) Primary spinal hydatid cyst in a patient with acquired immunodeficiency syndrome. Eur Spine J 20 Suppl 2: S235-238.

41. Erdincler P, Kaynar MY, Babuna O, Canbaz B (1997) The role of mebendazole in the surgical treatment of central nervous system hydatid disease. Br J Neurosurg 11: 116-120.

42. Essaadouni L, Jghaimi F, BenAli SA, Bouchti I, Kissani N (2009) Hydatid spinal cord compression revealing multivisceral hydatidosis. Clin Neurol Neurosurg 111: 918-919.

43. Fahl M, Haddad FS, Huballah M, Kana'an S, Husheimi I, et al. (1994) Magnetic resonance imaging in intradural and extradural spinal echinococcosis. Clin Imaging 18: 179-183.

44. Fares Y, Khazim R, El Zaatari MM, Haddad GF, Barnes PR (2003) Spinal hydatid disease and its neurological complications. Scand J Infect Dis 35: 394-396.

45. Fenyes G, Ladvanszky C (1977) [Echinococci of the spinal canal]. Zentralbl Neurochir 38: 157-164.

46. Ferrandez HD, Gomez-Castresana F, Lopez-Duran L, Mata P, Brandau D, et al. (1978) Osseous hydatidosis. J Bone Joint Surg Am 60: 685-690.

47. Ferris BD, Scott JE, Uttley D (1986) Hydatid disease of the cervical spine. Clin Orthop Relat Res: 174-177.

48. Fiennes AG, Thomas DG (1982) Combined medical and surgical treatment of spinal hydatid disease: a case report. J Neurol Neurosurg Psychiatry 45: 927-930.

49. Fitzpatrick SC (1965) Hydatid Disease of the Lumbar Vertebrae; Report of a Case. J Bone Joint Surg Br 47: 286-291.

50. Fkih L, Boussoffara L, Bedoui SA, Saad S, Belhabib D, et al. (2009) [Echinococcosis of the rib with epidural extension]. Rev Pneumol Clin 65: 169-172.

51. Garcia-Vicuna R, Carvajal I, Ortiz-Garcia A, Lopez-Robledillo JC, Laffon A, et al. (2000) Primary solitary Echinococcosis in cervical spine. Postsurgical successful outcome after long-term albendazole treatment. Spine (Phila Pa 1976) 25: 520-523.

52. Gelabert-Gonzalez M (2000) [Spinal cord compression due to the vertebral hydatid cyst]. Rev Neurol 30: 654-655.

53. Gezgin S, Sanal HT (2012) "Water-lily sign" as a rare finding of spinal hydatidosis. Spine J.

54. Gopal N, Chauhan S, Yogesh N (2007) Primary spinal extradural hydatid cyst causing spinal cord compression. Indian J Orthop 41: 76-78.

55. Govender TS, Aslam M, Parbhoo A, Corr P (2000) Hydatid disease of the spine. A long-term followup after surgical treatment. Clin Orthop Relat Res: 143-147.

56. Gunecs M, Akdemir H, Tugcu B, Gunaldi O, Gumucs E, et al. (2009) Multiple intradural spinal hydatid disease: a case report and review of literature. Spine (Phila Pa 1976) 34: E346-350.

57. Gurelik M, Goksel HM, Nadir A (2002) Posterior mediastinal paravertebral hydatid cyst causing severe paraparesis. Br J Neurosurg 16: 605-606.

58. Hamdan TA (2012) Hydatid disease of the spine: a report on nine patients. Int Orthop 36: 427-432.

59. Hamdan TA, Al-Kaisy MA (2000) Dumbbell hydatid cyst of the spine: case report and review of the literature. Spine (Phila Pa 1976) 25: 1296-1299.

60. Hemama M, Lasseini A, Rifi L, Boutarbouch M, Derraz S, et al. (2011) A sacral hydatid cyst mimicking an anterior sacral meningocele. J Neurosurg Pediatr 8: 526-529.

61. Herrera A, Martinez AA, Rodriguez J (2005) Spinal hydatidosis. Spine (Phila Pa 1976) 30: 2439-2444.

62. Hilmani S, El Malki M, Bertal A, Achouri M, Sami A, et al. (2004) [Lumbar intradural hydatid cyst. Case report]. Neurochirurgie 50: 57-60.

63. Iplikcioglu AC, Kokes F, Bayar A, Doganay S, Buharali Z (1991) Spinal invasion of pulmonary hydatidosis: computed tomographic demonstration. Neurosurgery 29: 467-468.

64. Islekel S, Ersahin Y, Zileli M, Oktar N, Oner K, et al. (1998) Spinal hydatid disease. Spinal Cord 36: 166-170.

65. Islekel S, Zileli M, Ersahin Y (1998) Intradural spinal hydatid cysts. Eur Spine J 7: 162-164.

66. Jaiswal S, Jaiswal AK, Jain M, Behari S, Pandey R (2009) Primary spinal extradural hydatid cyst causing paraplegia. Indian J Pathol Microbiol 52: 432-433.

67. Jena A, Tripathi RP, Jain AK (1991) Primary spinal echinococcosis causing paraplegia: case report with MR and pathologic correlation. AJNR Am J Neuroradiol 12: 560.

68. Joshi N, Hernandez-Martinez A, Seijas-Vazquez R (2007) Primary sacral hydatid cyst. A case report. Acta Orthop Belg 73: 674-677.

69. Kabbaj-El Kouhen N, Dafiri R, el Ouahabi A, el Khamlichi A, Imani F (1999) [Isolated lumbar intradural hydatid cyst]. J Radiol 80: 147-149.

70. Kaen A, Lagares A, Perez-Nunez A, Rivas JJ, Ramos A, et al. (2009) Intradural extramedullary spinal hydatidosis: case report. Neurocirugia (Astur) 20: 282-287.

71. Kahilogullari G, Tuna H, Aydin Z, Colpan E, Egemen N (2005) Primary intradural extramedullary hydatid cyst. Am J Med Sci 329: 202-204.

72. Kalkan E, Cengiz SL, Cicek O, Erdi F, Baysefer A (2007) Primary spinal intradural extramedullary hydatid cyst in a child. J Spinal Cord Med 30: 297-300.

73. Kalkan E, Torun F, Erdi F, Baysefer A (2008) Primary lumbar vertebral hydatid cyst. J Clin Neurosci 15: 472-473.

74. Kaoutzanis M, Anagnostopoulos D, Apostolou A (1989) Hydatid disease affecting the vertebrae. Acta Neurochir (Wien) 98: 60-65.

75. Karadereler S, Orakdogen M, Kilic K, Ozdogan C (2002) Primary spinal extradural hydatid cyst in a child: case report and review of the literature. Eur Spine J 11: 500-503.

76. Karantanas AH, Paterakis K, Karavelis A (2003) Intervertebral disk hydatid cysts: MR imaging findings. AJR Am J Roentgenol 180: 1739-1740.

77. Karray S, Karray M, Zlitni M, Douik M (2004) Radical cure of vertebral hydatidosis. A case report. Acta Orthop Belg 70: 80-83.

78. Karray S, Zlitni M, Fowles JV, Zouari O, Slimane N, et al. (1990) Vertebral hydatidosis and paraplegia. J Bone Joint Surg Br 72: 84-88.

79. Karray S, Zlitni M, Karray M, Douik M, Sliman N, et al. (1993) Extensive vertebral hydatidosis. A study. Acta Orthop Belg 59: 100-105.

80. Kars HZ, Hekimoglu B, Cepoglu C (1990) Spinal epidural hydatid cyst: radiological and ultrasonographical workup of a case. Eur J Radiol 11: 212-214.

81. Karvounis PC, Singounas EG, Tsaprounis G (1977) Intradural spinal echinococcus simulating lumbar disc protrusion. Neurochirurgia (Stuttg) 20: 58-60.

82. Keller TM, Schweitzer JS, Helfend LK, Chappell T (1997) Treatment of progressive cervical spinal instability secondary to hydatid disease. A case report. Spine (Phila Pa 1976) 22: 915-919.

83. Khazim R, Fares Y, Heras-Palou C, Ruiz Barnes P (2003) Posterior decompression of spinal hydatidosis: long term results: Fundacion Jimenez Diaz, Madrid, Spain. Clin Neurol Neurosurg 105: 209-214.

84. Kilic D, Cangir AK, Bulut S, Akay H (2003) Hydatid cyst of the rib with intramedullary involvement. Acta Chir Belg 103: 334-335.

85. Kilic D, Erdogan B, Sener L, Sahin E, Caner H, et al. (2006) Unusual dumbbell tumours of the mediastinum and thoracic spine. J Clin Neurosci 13: 958-962.

86. Klouche K, Charlotte N, Kaaki M, Beraud JJ (1994) Coma and haemolysis after cetrimide washout of epidural hydatid cyst. Intensive Care Med 20: 613.

87. Kolsi M, Ghorbel M, Abdennadher M, Chabchoub I, Ben Mansour H, et al. (2005) [Costo-vertebral collection complicating multifocal echinococcosis: surgical drainage via an anterior approach]. Rev Mal Respir 22: 673-676.

88. Kotil K, Tari R, Savas Y (2010) Medical treatment of primary extradural solitary lumbar hydatid disease. J Clin Neurosci 17: 793-795.

89. Kotil K, Tatar Z, Bilge T (2007) Spinal hydatidosis accompanied by a secondary infection. Case report. J Neurosurg Spine 6: 585-590.

90. Kuremu RT, Khwa-Otsyula BO, Svanvik J, Bwombengi OS, Lelei LK, et al. (2002) Hydatid disease of the spine: case report. East Afr Med J 79: 165-166.

91. Lakhdar F, Arkha Y, Rifi L, Derraz S, El Ouahabi A, et al. (2009) Spinal intradural extramedullary hydatidosis: report of three cases. Neurosurgery 65: 372-376; discussion 376-377.

92. Lam KS, Faraj A, Mulholland RC, Finch RG (1997) Medical decompression of vertebral hydatidosis. Spine (Phila Pa 1976) 22: 2050-2055.

93. Lath R, Ratnam BG, Ranjan A (2007) Diagnosis and treatment of multiple hydatid cysts at the craniovertebral junction. Case report. J Neurosurg Spine 6: 174-177.

94. Law WB (1965) Extradural Spinal Hydatid Cyst: A Case Report. Aust N Z J Surg 34: 215-217.

95. Layadi F, Boubrik M, Ait El Qadi A, Ait Benali S (2005) [Primary sacral epidural hydatid cyst: a case report]. J Radiol 86: 1040-1042.

96. Levack B, Kernohan J, Edgar MA, Ransford AO (1986) Observations on the current and future surgical management of hydatid disease affecting the vertebrae. Spine (Phila Pa 1976) 11: 583-590.

97. Ley A, Jr., Marti A (1970) Intramedullary hydatid cyst. Case report. J Neurosurg 33: 457-459.

98. Limaiem F, Bellil S, Bellil K, Chelly I, Mekni A, et al. (2010) Primary hydatidosis of the central nervous system: a retrospective study of 39 Tunisian cases. Clin Neurol Neurosurg 112: 23-28.

99. Logroscino CA, Pola E, Pola R, Tamburrelli FC (2005) Hydatid cyst of the spine. Lancet Infect Dis 5: 732.

100. Maalej S, Belhabib D, Hantous S, Fenniche S, Ammar A, et al. (2003) [Costo-vertebral hydatid disease: the role of MRI]. Rev Mal Respir 20: 614-617.

101. Mahi M, Amil T, Chaouir S, Hanine A, Benameur M (2001) [Imaging of a historical case of vertebral hydatidosis]. J Neuroradiol 28: 244-248.

102. Maiuri F, Iaconetta G, Benvenuti D, Rendano F, Serra LL (1993) Hydatid cyst of the lumbosacral spine with large pelvic mass. Acta Neurol (Napoli) 15: 215-221.

103. Malloch JD (1965) Hydatid Disease of the Spine. Br Med J 1: 633.

104. Mathuriya SN, Arora OP, Khosla VK, Prabhakar SK, Chopra JS, et al. (1985) Infected intradural hydatid cyst at foramen magnum. A case report. Clin Neurol Neurosurg 87: 283-286.

105. McNeur JC, Dudley HA (1973) Hydatid disease of the spine. J R Coll Surg Edinb 18: 76-78.

106. Medjek L, Zenini S, Hammoum S, Hartani M (1991) [Intradural hydatidosis of the thoracic spine. Apropos of a case]. Ann Radiol (Paris) 34: 251-255.

107. Midyat L, Gokce S, Onder A, Ozdemir Y, Mursalov G, et al. (2009) A very rare cause of childhood paraparesis: primary intradural extramedullary spinal hydatid cyst. Pediatr Infect Dis J 28: 754-755.

108. Mikhael MA, Ciric IS, Tarkington JA (1985) MR imaging in spinal echinococcosis. J Comput Assist Tomogr 9: 398-400.

109. Miller LK, Miller JW, Fry WA (1981) Paraspinous mass in a greek woman. Chest 80: 741-742.

110. Moharamzad Y, Kharazi HH, Shobeiri E, Farzanegan G, Hashemi F, et al. (2008) Disseminated intraspinal hydatid disease. J Neurosurg Spine 8: 490-493.

111. Monajati A, Zarrabi M, Shaffi M (1976) Primary costal echinococcosis with spine involvement. Br J Radiol 49: 555-556.

112. Morshed AA (1977) Hydatid disease of the spine. Neurochirurgia (Stuttg) 20: 211-215.

113. Mrabet D, Rekik S, Khiari H, Mizouni H, Meddeb N, et al. (2011) Back pain caused by a pseudo-tumorous vertebral collapse: atypical presentation of primary vertebral hydatidosis. BMJ Case Rep 2011.

114. Navarro Artiles G (1972) [Recurrent and progressive paraplegia due to D1-D9 multiple extradural hydatidosis, operated on 6 times]. Rev Clin Esp 125: 461-464.

115. Ndondo AP, Fieggen G, Wilmshurst JM (2003) Hydatid disease of the spine in South African children. J Child Neurol 18: 343-346.

116. Onbas O, Kantarci M, Alper F, Sekmenli N, Okur A (2004) Spinal widespread intradural extramedullary hydatidosis. Neuroradiology 46: 310-312.

117. Ouma JR (2006) Echinococcosis--a rare spinal disorder. S Afr Med J 96: 680, 682.

118. Oumerzouk J, Hssaini Y, Qamouss O, Baalal H, Bourazza A (2012) [Multifocal hydatidosis with extended vertebral and spinal cord localization.]. Presse Med.

119. Ozcan HN, Kara M, Azboy D, Ozcakar L (2009) Hydatid cyst disease of the spine. Am J Phys Med Rehabil 88: 435.

120. Ozdemir HM, Ogun TC, Tasbas B (2004) A lasting solution is hard to achieve in primary hydatid disease of the spine: long-term results and an overview. Spine (Phila Pa 1976) 29: 932-937.

121. Ozdemir O, Calisaneller T, Yildirim E, Altinors N (2011) Percutaneous CT-guided treatment of recurrent spinal cyst hydatid. Turk Neurosurg 21: 685-687.

122. Ozer AF, Ozek MM, Pamir MN, Erzen C (1993) Magnetic resonance imaging in the diagnosis of spinal hydatid cyst disease. Case report. Paraplegia 31: 338-340.

123. Pamir MN, Akalan N, Ozgen T, Erbengi A (1984) Spinal hydatid cysts. Surg Neurol 21: 53-57.

124. Panahi S (1983) [Echinococcus cysticus in the spinal canal in spinal cord compression]. Dtsch Med Wochenschr 108: 76.

125. Pandey M, Chaudhari MP (1997) Primary hydatid cyst of sacral spinal canal: case report. Neurosurgery 40: 407-409.

126. Papakonstantinou O, Athanassopoulou A, Passomenos D, Kalogeropoulos I, Balanika A, et al. (2011) Recurrent vertebral hydatid disease: spectrum of MR imaging features. Singapore Med J 52: 440-445.

127. Parvaresh M, Moin H, Miles JB (1996) Dumbbell hydatid cyst of the spine. Br J Neurosurg 10: 211-213.

128. Pasaoglu E, Boyacigil S, Damgaci L, Tokoglu F, Soydinc P, et al. (1997) Vertebral hydatid disease. Australas Radiol 41: 188-189.

129. Pau A, Cossu M, Viale ES, Siccardi D, Turtas S, et al. (1986) [Echinococcosis of the spine]. Zentralbl Neurochir 47: 316-321.

130. Perani D, Scotti G, Scialfa G, D'Angelo V (1983) Hydatid disease of spine. A case report. J Neurosurg Sci 27: 55-58.

131. Pluchino F, Lodrini S (1981) Multiple primitive epidural spinal hydatid cysts: case report. Acta Neurochir (Wien) 59: 257-262.

132. Poggianti G, Ferrari FS, Risso G, Belcapo L, Tiribocchi A (1997) [Pulmonary hydatidosis with spinal involvement. A case report]. Radiol Med 94: 683-685.

133. Porat S, Robin GC, Wertheim G (1984) Hydatid disease of the spine causing paraplegia. The combined treatment by surgical drainage and mebendazole: a case report. Spine (Phila Pa 1976) 9: 648-653.

134. Prabhakar MM, Thakker T (2006) Anterior decompression for cervicothoracic pathology: A study of 14 patients. J Spinal Cord Med 29: 163-166.

135. Psomopoulos N, Vlachakis T, Balaroutsos C, Voulgaris J, Delikaris P, et al. (1976) [Bone echinococcosis. Apropos of a case of hydatid cyst of the spine]. J Chir (Paris) 111: 207-210.

136. Pushparaj K, Sundararajan M, Madeswaran K, Ambalavanan S (2001) Primary spinal intradural hydatid cyst--a short report. Neurol India 49: 203-204.

137. Ranganadham P, Dinakar I, Sundaram C, Ratnakar KS, Vivekananda T (1990) Posterior mediastinal paravertebral hydatid cyst presenting as spinal compression. A case report. Clin Neurol Neurosurg 92: 149-151.

138. Rao S, Parikh S, Kerr R (1991) Echinococcal infestation of the spine in North America. Clin Orthop Relat Res: 164-169.

139. Rkain H, Bahiri R, Benbouazza K, Hajjaj-Hassouni N (2007) An extensive vertebral hydatidosis revealed by a lumbosciatica. Clin Rheumatol 26: 1386-1388.

140. Ruelle A, Boccardo M, Lasio G, Severi P (1985) [Primary vertebral hydatidosis. Case report and brief review of the literature]. Riv Neurol 55: 332-337.

141. Rumana M, Mahadevan A, Nayil Khurshid M, Kovoor JM, Yasha TC, et al. (2006) Cestode parasitic infestation: intracranial and spinal hydatid disease--a clinicopathological study of 29 cases from South India. Clin Neuropathol 25: 98-104.

142. Sacerdoti S, Stabile F (1988) [A case of paravertebral Echinococcus cysts communicating with the intraspinal epidural space]. Radiol Med 75: 243-245.

143. Salduz A, Koyuncu LO, Dikici F, Talu U (2009) [Long-term result of treatment for paraspinal and extradural hydatid cyst: a case report]. Acta Orthop Traumatol Turc 43: 267-271.

144. Samadian M, Alavi E, Sharifi G, Rezaee O, Faramarzi F (2010) Extension of echinococcal spinal infestation extra- and intradurally after a decade of extinction. J Neurosurg Sci 54: 143-148.

145. San Martin Sanchez L, Lopez Zafra JJ, de la Riva Aguilar A, Oliva Alonso JM, Donnay Brisa G (1980) [Spinal sub-dural hydatidosis. A report on one case (author's transl)]. Neurochirurgie 26: 235-238.

146. Santavirta S, Valtonen M, Patiala H, Saarinen O, Konttinen YT (2000) Disseminated hydatid disease causing paraplegia and destruction of the hip. Arch Orthop Trauma Surg 120: 118-120.

147. Sapkas GS, Machinis TG, Chloros GD, Fountas KN, Themistocleous GS, et al. (2006) Spinal hydatid disease, a rare but existent pathological entity: case report and review of the literature. South Med J 99: 178-183.

148. Sapkas GS, Papagelopoulos PJ, Stathakopoulos DP, Babis GC, Tzagarakis GP, et al. (2002) Recurrent paraplegia caused by spinal echinococcosis. Orthopedics 25: 1087-1088.

149. Sapkas GS, Stathakopoulos DP, Babis GC, Tsarouchas JK (1998) Hydatid disease of bones and joints. 8 cases followed for 4-16 years. Acta Orthop Scand 69: 89-94.

150. Sapunar J, Gambini L (1993) [Vertebral hydatidosis with extra- and intra-rachidian hydatid ossifluent pseudoabscess]. Bol Chil Parasitol 48: 18-25.

151. Saracino A, Scotto G, Palumbo E, Cibelli D, Tartaglia A, et al. (2005) Multiple recurrences of vertebral hydatidosis: a case report. New Microbiol 28: 271-275.

152. Sasani M, Ozer AF (2009) Spontaneous drainage of an asymptomatic recurrent hydatid cyst of the sacrum. Spine (Phila Pa 1976) 34: E269-271.

153. Savini R, Figus E, Maggi G, Rizqallah Y (1984) [Use of mebendazole in association with the surgical therapy of vertebral hydatidosis]. Chir Organi Mov 69: 135-142.

154. Scarlata F, Giordano S, Saporito L, Marasa L, Li Pani G, et al. (2011) Cystic hydatidosis: a rare case of spine localization. Infez Med 19: 39-41.

155. Schnepper GD, Johnson WD (2004) Recurrent spinal hydatidosis in North America. Case report and review of the literature. Neurosurg Focus 17: E8.

156. Secer HI, Anik I, Celik E, Daneyemez MK, Gonul E (2008) Spinal hydatid cyst mimicking arachnoid cyst on magnetic resonance imaging. J Spinal Cord Med 31: 106-108.

157. Semlali S, Nassar I, Cisse A, El-Gueddari FZ, Imani F (2004) [Cervical hydatid disease with retropharyngeal involvement: a case report]. J Radiol 85: 51-53.

158. Senar Calderon A, Barriendos Villagrasa J, Sanchez Garcia F, Badorrey Martin MI (1996) [Pulmonary hydatidosis as a cause of spinal cord compression]. Arch Bronconeumol 32: 108-110.

159. Sener RN, Calli C, Kitis O, Yalman O (2001) Multiple, primary spinal-paraspinal hydatid cysts. Eur Radiol 11: 2314-2316.

160. Sengul G, Kadioglu HH, Kayaoglu CR, Aktas S, Akar A, et al. (2008) Treatment of spinal hydatid disease: a single center experience. J Clin Neurosci 15: 507-510.

161. Senoglu M, Bulbuloglu E, Demirpolat G, Altun I, Celik M (2009) Combined anterior and posterior approach for sacral/retroperitoneal hydatid cyst disease: case report. Turk Neurosurg 19: 428-432.

162. Sharma A, Kashyap V, Abraham J, S K (1981) Intradural hydatid cysts of the spinal cord. Surgical Neurology 16: 235-237.

163. Sharma NK, Chitkara N, Bakshi N, Gupta P (2003) Primary spinal extradural hydatid cyst. Neurol India 51: 89-90.

164. Shukla SK, Sharma V, Singh K, Trivedi A (2010) Primary lumbosacral intradural hydatid cyst in a child. J Neurosci Rural Pract 1: 109-111.

165. Singh P, Shankar S, Sharma BS, Nk V, Khandelwal N, et al. (2000) MRI in sacral echinococcosis. J Comput Assist Tomogr 24: 176-177.

166. Song X, Liu D, Wen H (2007) Diagnostic pitfalls of spinal echinococcosis. J Spinal Disord Tech 20: 180-185.

167. Spektor S, Gomori JM, Beni-Adani L, Constantini S (1997) Spinal echinococcal cyst: treatment using computerized tomography-guided needle aspiration and hypertonic saline irrigation. Case report. J Neurosurg 87: 464-467.

168. Spies C, Weisskopf M, Ohnsorge JA (2008) [Intraspinal echinococcosis within the lumbar spine of an 18-year-old male patient]. Z Orthop Unfall 146: 463-467.

169. Stewart GR, Loewenthal J (1967) Vertebral hydatidosis. Aust N Z J Surg 36: 175-183.

170. Sudo H, Minami A (2010) Neurological picture. A widespread echinococcosis of the spine. J Neurol Neurosurg Psychiatry 81: 892.

171. Suri S, Singh I, Gulati DR, Sodhi JS (1978) Hydatid cyst causing spinal compression. Paraplegia 15: 333-337.

172. Tabak O, Yilmaz M, Oz B, Ozaras R, Erdincler P, et al. (2007) A vertebral hydatid cyst infection mimicking tuberculous spondylodiscitis. J Infect Chemother 13: 180-182.

173. Tammam A, Crockard HA (1997) Quadriplegia with cervical cord compression. Postgrad Med J 73: 381-382.

174. Tapia EO, Vidal TA, Bellolio JE, Roa SJ (2010) [Bone hydatidosis: report of five patients and review of the literature]. Rev Med Chil 138: 1414-1421.

175. Tekkok IH, Benli K (1993) Primary spinal extradural hydatid disease: report of a case with magnetic resonance characteristics and pathological correlation. Neurosurgery 33: 320-323; discussion 323.

176. Thaler M, Gabl M, Lechner R, Gstottner M, Bach CM (2010) Severe kyphoscoliosis after primary Echinococcus granulosus infection of the spine. Eur Spine J 19: 1415-1422.

177. Tugcu B, Gunaldi O, Gunes M, Guler AK, Adilay U, et al. (2008) Hydatid cysts in uncommon locations in the same patient: simultaneous cardiac and spinal involvement. Minim Invasive Neurosurg 51: 234-236.

178. Turan Suslu H, Cecen A, Karaaslan A, Borekci A, Bozbuga M (2009) Primary spinal hydatid disease. Turk Neurosurg 19: 186-188.

179. Turtas S, Viale ES, Pau A (1980) Long-term results of surgery for hydatid disease of the spine. Surg Neurol 13: 468-470.

180. Viljoen H, Crane J (2008) Hydatid disease of the spine. Spine (Phila Pa 1976) 33: 2479-2480.

181. von Sinner WN, Akhtar M (1994) Case report 833: Primary spinal echinococcosis (Echinococcus granulosus) of lumbosacral spine with destruction of the left pedicles of L3-5 and extension of a large paraspinal cystic mass into the spinal canal. Skeletal Radiol 23: 220-223.

182. Wang Y, Geng D, Zhu G, Du G (2009) Primary spinal extradural hydatid cyst associated with acute bleeding. N Am J Med Sci 1: 78-81.

183. Wani MA, Taheri SA, Babu ML, Ahangar GA, Wani H (1989) Primary spinal extradural hydatid cyst. Neurosurgery 24: 631-632.

184. Xin L, Wang Z, Fan S (2009) Magnetic resonance imaging and computerised tomography findings in an intraspinal extradural hydatid cyst mimicking tuberculous spondylitis: a case report. Cases J 2: 7109.

185. Yegen C, Ozer AF, Aktan AO, Yalin R (1993) Sacrococcygeal hydatid cyst: another entity in the differential diagnosis of sacrococcygeal chordoma. Case report. Paraplegia 31: 479-481.

186. Younan T, Sfeir S, Kheir C, Slaba S (2009) [Infectious spondylodiscitis as a presentation for paravertebral hydatid cyst]. J Radiol 90: 507-509.

187. Zaalouni I, Ouertatani M, Meherzi MH, Ben Hamida MK, Rbai H, et al. (2010) [Costovertebral echinococcosis: a case report and review of the literature]. Rev Med Interne 31: 69-71.

188. Zhang Z, Li F, Zhao G, Sun T (2012) "Bunch of grapes" on the spine-spinal hydatidosis. Braz J Infect Dis 16: 313-314.

189. Zheng XF, Sheng WB (2011) Osteolytic expansile lesions in lumbosacral hydatid disease. Spine J 11: 1076-1077.

**References of excluded publications**

1. Atanasov A (1965) [A case of multiple echinococcal cysts of the lung, liver and lumbosacral spinal region]. Khirurgiia (Sofiia) 18: 595-596.

2. Pierini LD, Migliardi CD, Veppo AA (1965) [Hydatid compression of the cervical cord]. Rev Asoc Med Argent 79: 510-514.

3. Vengsarkar US, Abraham J (1965) Hydatid disease of the spine. A case report. J Postgrad Med 11: 133-136.

4. Catena E, Bariffi F, Del Bono M (1966) [Morphological changes of the bronchi in some chronic bronchopneumopathies]. Arch Tisiol Mal Appar Respir 21: 453-463.

5. Ferkovic M (1966) [Apropos of a case of echinococcosis of the cauda equina]. Lijec Vjesn 88: 161-165.

6. Ferrand J, Chitour S, Castillon A (1966) ["Solitary" epidural hydatid cyst]. J Chir (Paris) 91: 191-197.

7. Milenkovic P, Stula D (1966) [Echinococcosis of the central nervous system]. Srp Arh Celok Lek 94: 163-167.

8. Samiy E (1966) Involvement of the central nervous system by hydatid cyst. Br J Clin Pract 20: 143-151.

9. Borne G, Hamidou B (1967) [Spinal cord compression by vertebral echinococcosis of extraspinal origin. Reflections apropos of 2 cases]. Bull Soc Pathol Exot Filiales 60: 282-291.

10. Lus EA, Fedorov BN, Khoteeva AF (1967) [Echinococcosis of the cervical spine]. Vestn Khir Im I I Grek 98: 115-116.

11. Milton GW, Conrad P, Claffey TJ (1967) Gastrostomy in the treatment of advanced sepsis: a suggestion. Aust N Z J Surg 37: 79-80.

12. Neubauer W, Wiandt J (1967) [Contribution to the diagnosis and surgical management of echinococcosis]. Chirurg 38: 229-232.

13. Zmerli S, Abada M, Court B (1967) [Echinococcosis of the sacrum with a urologic mask]. J Chir (Paris) 93: 573-578.

14. Aulong C, Franquet F, Benchekroun A, Blondel P (1968) [3 cases of osseous echinococcosis]. Mem Acad Chir (Paris) 94: 237-244.

15. Durov MF (1968) [A case of echinococcosis of the cervico-thoracic region of the spine with compression of the spinal cord]. Ortop Travmatol Protez 29: 57-59.

16. Gokay H (1968) [Hydatid cyst of the vertebrae (apropos of 10 cases)]. Turk Tip Cemiy Mecm 34: 165-178.

17. Hernandez E, Llanos A (1968) [Vertebral hydatidosis. Findings on 7 cases]. Bol Chil Parasitol 23: 129-130.

18. Mineiro JD (1968) [Destructive secondary processes of the human spine. II]. Arq Patol 40: 81-98.

19. Demartin F (1969) [Sacro-coccigeal echinococcosis]. Chir Organi Mov 57: 517-527.

20. Dressler S, Haring R, Klems H (1969) [Clinical findings and therapy of abdominal echinococcosis]. Zentralbl Chir 94: 772-781.

21. Ottolenghi CE (1969) Aspiration biopsy of the spine. Technique for the thoracic spine and results of twenty-eight biopsies in this region and over-all results of 1050 biopsies of other spinal segments. J Bone Joint Surg Am 51: 1531-1544.

22. Riddell RJ (1969) Hydatid disease of the spine with subdural spread: case report. Pathology 1: 129-131.

23. Suleimanov I (1969) [A case of echinococcosis of the thoracic spine with spinal cord compression]. Ortop Travmatol Protez 30: 79-81.

24. Balasubramaniam V, Ramanujam PB, Ramamurthi B (1970) Hydatid disease of the nervous system. Neurol India 18: Suppl 1:92-95.

25. Szava J, Geambazu E, Vanky F, Cozmuta M (1970) Vertebral echinococcosis. Rom Med Rev 14: 47-54.

26. Arcan P, Ionescu E, Dragan G, Borzan N (1971) [Unusual localizations of hydatidosis. Intraspinal cysts]. Neurol Psihiatr Neurochir 16: 37-44.

27. Moldaver-Cherdzhieva TI, Raillo IV, Ostrovskii EA (1971) [Fistulography with the use of a vacuum device]. Probl Tuberk 49: 48-51.

28. Slim MS, Khayat G, Nasr AT, Jidejian YD (1971) Hydatid disease in childhood. J Pediatr Surg 6: 440-448.

29. Filatova AV, Zhdanova AV (1972) [Diagnostic error in the diagnosing of echinococcosis of the spine]. Ortop Travmatol Protez 33: 71-72.

30. Lievre JA, Camus JP, Darcy M, Pradat P (1972) [Total spondylectomy (extra-ligamental exeresis of a vertebra) (2 cases)]. Ann Med Interne (Paris) 123: 887-894.

31. Navarro Artiles G, Crespo-Lopez V, Matias Riu J (1972) [Various differences between cerebral and spinal cord pathology]. Rev Clin Esp 124: 167-170.

32. Schantz PM (1972) [Localization of hydatidosis in the central nervous system]. Bol Oficina Sanit Panam 73: 198-202.

33. Carcassonne M, Aubrespy P, Dor V, Choux M (1973) Hydatid cysts in childhood. Prog Pediatr Surg 5: 1-35.

34. Mempel E, Grochowski W (1973) [Multiple echinococcal cysts in posterior cranial fossa and spinal cord treated surgically]. Neurol Neurochir Pol 7: 741-744.

35. Bouvier M, Lejeune E, Jeanneret J, Amourdedieu J, Monnet M (1974) [The solitary epidural hydatid cyst]. Rev Rhum Mal Osteoartic 41: 173-177.

36. Fowler ME, Silverman S, Schulz TA, Baker N (1974) Osseous hydatidosis in a patas monkey. J Am Vet Med Assoc 165: 840-844.

37. Getaz P, Handler L, Jacobs P, Tunley I (1974) Osteosclerotic myeloma with peripheral neuropathy. S Afr Med J 48: 1246-1250.

38. Janecka V, Bruna J (1974) [Bone echinococcosis (author's transl)]. Acta Chir Orthop Traumatol Cech 41: 239-243.

39. Procek J (1974) [Hydatic cysts of the spine (author's transl)]. Acta Chir Orthop Traumatol Cech 41: 419-422.

40. Sathyanarayana K, Sunder PS, Reddy DR, Rao JJ, Rajan KH, et al. (1974) Sinal hydatid disease--a case report. J Assoc Physicians India 22: 927-929.

41. Abelanet R, Forest M, Palangie A, Meary R, Tomeno B, et al. (1975) [Osseous echinococcosis. Apropos of 6 anatomo-clinical observations]. Ann Anat Pathol (Paris) 20: 133-148.

42. Carrea R, Dowling E, Jr., Guevara JA (1975) Surgical treatment of hydatid cysts of the central nervous system in the pediatric age (Dowling's technique). Childs Brain 1: 4-21.

43. Mosavy SH, Sajadieh V, Vakhshuri P (1975) Echinococcal cyst beyond the pulmonary sieve. Surgery 77: 194-200.

44. Apt WL, Fierro JL, Calderon C, Perez C, Mujica P (1976) Vertebral hydatid disease. Clinical experience with 27 cases. J Neurosurg 44: 72-76.

45. Barrucand D, Hermo J, Ouarzazi A, Schmitt J, Schmidt C (1976) [The neurosurgical aspects of hydatid cysts]. Rev Otoneuroophtalmol 48: 291-311.

46. Elies W, Pirschel J (1976) [Echinococcus cysticus of the lumbosacral transitional area with para- and intravertebral localization]. Rofo 124: 187-188.

47. Louis R, Casanova J, Baffert M (1976) [Surgical technics in tumors of the spine]. Rev Chir Orthop Reparatrice Appar Mot 62: 57-70.

48. Nourmand A (1976) Hydatid cysts in children and youths. Am J Trop Med Hyg 25: 845-847.

49. Gharbi HA, Cheikh MB, Hamaza R, Jeddi M, Hamza B, et al. (1977) Rare sites of hydatid disease in children. Ann Radiol (Paris) 20: 151-157.

50. Goddard RM (1978) Hydatid disease of spine. Proc Mine Med Off Assoc SA 57: 78.

51. Savini R, Capelli A (1978) Vertebral localization of echinococcosis (case report with medullary compression). Ital J Orthop Traumatol 4: 99-104.

52. Cheng GY (1979) [Hydatid cysts of the spinal canal (author's transl)]. Zhonghua Fang She Xue Za Zhi 13: 48-49.

53. Geissl G (1979) [Tuberculous spondylitis--Pott's disease]. MMW Munch Med Wochenschr 121: 39.

54. Nyul-Toth P, Risko T, Molnar M (1979) [Echinococcus in the bone (author's transl)]. Magy Traumatol Orthop Helyreallito Seb 22: 40-46.

55. Wildbolz A (1979) [Diagnostic errors due to unilateral psychiatric or unilateral somatic appreciation (author's transl)]. Schweiz Rundsch Med Prax 68: 1482-1487.

56. Kaufman DM, Kaplan JG, Litman N (1980) Infectious agents in spinal epidural abscesses. Neurology 30: 844-850.

57. Le Beau J, Corcos A (1980) [Neurosurgical observations of central nervous system parasitic diseases]. Sem Hop 56: 613-618.

58. Salva Verd A, Zudaire Bergera JJ, Berian Polo JM, Pomar Moya-Prats P (1980) [Retrovesical hydatidosis]. Actas Urol Esp 4: 335-338.

59. Escudero Barrilero A, Romero Maroto J, Mayayo Dehesa T, Maganto Pavon E, Perales Cabanas L, et al. (1981) [Value of translumbar puncture in the diagnosis of renal hydatid cyst]. Rev Clin Esp 162: 179-187.

60. Giordano GB, Cerisoli M, Bernardi B (1982) Hydatid cysts of the spine. J Comput Assist Tomogr 6: 408-409.

61. Gyuris J, Fenyes G, Ladvanszki C (1982) [Echinococcosis in the lower section of the spinal canal]. Orv Hetil 123: 2361-2363.

62. Honma K, Sasano N, Andoh N, Iwai K (1982) Hepatic alveolar echinococcosis invading pancreas, vertebrae, and spinal cord. Hum Pathol 13: 944-946.

63. Meyer E, Adar R (1982) Echinococcosis of the spine and retroperitoneum: case report and review of the literature. Henry Ford Hosp Med J 30: 11-13.

64. Bagchi AK (1983) Infections and infestations of the central nervous system in India. A review. Neurosurg Rev 6: 93-101.

65. Cardona JM, Gine J, Flores X, Algara C, Ballester J (1983) [2 cases of vertebral hydatidosis treated by the association of surgery and mebendazole]. Rev Chir Orthop Reparatrice Appar Mot 69: 69-74.

66. Gaucher A, Vinet E, Pere P, Plenat F, Ethgen D, et al. (1983) [Alveolar echinococcosis with spinal localization]. Presse Med 12: 1366.

67. Toumbouras M, Sbarounis CN, Semoglou C, Sagadamis A, Lazarides D (1983) [Rare primary localizations of Echinococcus cysticus]. Zentralbl Chir 108: 654-659.

68. Yadav SS, Nagabhushana KR (1983) Hydated disease of the spine with paraplegia. (Report of a case). Indian J Med Sci 37: 30-32.

69. Konjhodzic F (1984) [Spinal echinococcosis]. Med Arh 38: 101-103.

70. Schubiger O, Valavanis A, Hollmann J (1984) Computed tomography of the intervertebral foramen. Neuroradiology 26: 439-444.

71. Adler CP (1985) [Spondylitis--spondylodiscitis. Pathologico-anatomical morphology and diagnostic problems]. Radiologe 25: 291-298.

72. Fournier JP, Marty P, Bernard E, Leloire P, Dellamonica P, et al. (1985) [Spinal hydatidosis treated by albendazole. A propos of 2 cases]. Pathol Biol (Paris) 33: 611-613.

73. Rami-Porta R, Bravo-Bravo JL, Aroca-Gonzalez MJ, Alix-Treuba A, Serrano-Munoz F (1985) Tumours and pseudotumours of the chest wall. Scand J Thorac Cardiovasc Surg 19: 97-103.

74. Rong SH, Nie ZQ (1985) Hydatid disease of bone. Clin Radiol 36: 301-305.

75. Tazi Z, Boujida N, Hamdouch N, Boukhrissi N (1985) [Vertebral and spinal cord hydatidosis. Contribution of radiology and x-ray computed tomography. Apropos of 36 cases]. J Radiol 66: 183-188.

76. Clements R, Gravelle IH (1986) Radiological appearances of hydatid disease in Wales. Postgrad Med J 62: 167-173.

77. Dellamonica P, Le Fichoux Y (1986) [Spinal hydatidosis. Failure of albendazole]. Pathol Biol (Paris) 34: 295-296.

78. Esposito R, Orlando G, Crocchiolo P, Lazzarin A, D'Arminio Monforte A, et al. (1986) Albendazole in the treatment of cystic hydatid disease. Boll Ist Sieroter Milan 65: 516-522.

79. Stula D, Liverani F, Gratzl O (1986) [Development, symptomatology and treatment of "animal" and "plant" parasites of the central nervous system]. Schweiz Arch Neurol Psychiatr 137: 15-23.

80. Claudon M, Bracard S, Plenat F, Regent D, Bernadac P, et al. (1987) Spinal involvement in alveolar echinococcosis: assessment of two cases. Radiology 162: 571-572.

81. Oueslati A, Hamza HA, Fodha M, Ladeb F, Kammoun H, et al. (1987) [Bone echinococcosis. Apropos of 4 cases]. Tunis Med 65: 291-297.

82. Arboix A, Tordesillas C, Oliver B, Molet J, Bartumeus F, et al. (1988) [Growth of a cerebral hydatid cyst evaluated by CT study]. Neurologia 3: 31-34.

83. Lassale B, Gayet B, Antonietti P, Deburge A (1988) [Anterior medial approach in vertebrectomy of L1 and L2 with primary control of the aorta and the inferior vena cava]. Rev Chir Orthop Reparatrice Appar Mot 74 Suppl 2: 67-70.

84. Weber M, Vespignani H, Jacquier P, Gerard A, Claudon P, et al. (1988) [Neurological manifestations of alveolar echinococcosis]. Rev Neurol (Paris) 144: 104-112.

85. Zhu ZC (1988) [Diagnosis and treatment of intraspinal hydatidosis]. Zhonghua Wai Ke Za Zhi 26: 170-171, 191-172.

86. Argenson C, Griffet J, Lacour C, Arcamone H, Lovet J, et al. (1989) [Vertebral hydatid cyst. Apropos of 2 cases]. Rev Chir Orthop Reparatrice Appar Mot 75: 267-270.

87. Custovic K, Skljarevski V (1989) [Complicated echinococcosis in a rare location--case report]. Acta Chir Iugosl 36 Suppl 2: 624-625.

88. Metaizeau JP, Czorny A, Miahle C, Kuhnast M, Prevot J (1989) [Use of vascularized bone grafts in surgery of the spine. Apropos of 6 cases]. Rev Chir Orthop Reparatrice Appar Mot 75: 166-171.

89. Rieber A, Brambs HJ, Friedl P (1989) [CT in echinococcosis of the lumbar spine and paravertebral structures]. Rofo 151: 379-380.

90. Salvati M, Tossini A, Caruso R, Artizzu S, Ramundo Orlando E, et al. (1989) [Spinal hydatidosis: state of current knowledge on its lesions and presentation of an unusual clinical case]. G Chir 10: 727-729.

91. Tavoulari-Chrysophaki KG, Emmanouilidou AJ (1989) Primary diagnosis of intraspinal echinococcosis by cytologic examination of cyst fluid. Acta Cytol 33: 138-140.

92. Vetska P, Tsekov K, Nedev N (1989) [Intramedullary echinococcosis in a 9-year-old child]. Zh Vopr Neirokhir Im N N Burdenko: 55-56.

93. Vujovic R, Pajevic N, Vukovic N, Davidovic R, Perovic M, et al. (1989) [Echinococcosis of the spinal column]. Acta Chir Iugosl 36 Suppl 2: 621-624.

94. Wrazidlo W, Rieber A, Schneider S, Brambs HJ, Friedel P (1989) [A rare manifestation of Echinococcus cysticus in CT and magnetic resonance tomography]. Digitale Bilddiagn 9: 102-104.

95. Dernevik L, Larsson S (1990) Management of dumbbell tumours. Reports of seven cases. Scand J Thorac Cardiovasc Surg 24: 47-51.

96. Manojlovic D, Stijelja B, Milovic N (1990) [Echinococcal renal cyst]. Srp Arh Celok Lek 118: 227-229.

97. Martin Garcia V, Arrazola Garcia J, Ganado Diaz T, Yanguela Rodilla F, Alberti Jaume E (1990) [A 60-year-old man with a palpable abdominal mass and constipation]. Rev Clin Esp 187: 249-251.

98. Richards KS, Morris DL (1990) Effect of albendazole on human hydatid cysts: an ultrastructural study. HPB Surg 2: 105-112; discussion 112-103.

99. Rosin VS (1990) [Echinococcosis of the spinal canal]. Klin Med (Mosk) 68: 60-62.

100. Torricelli P, Martinelli C, Biagini R, Ruggieri P, De Cristofaro R (1990) Radiographic and computed tomographic findings in hydatid disease of bone. Skeletal Radiol 19: 435-439.

101. Garmendia Larrea JC, SanRoma Ortueta I, Rodriguez Andres JA, Garrido Rivas C, Lopez Garcia JA, et al. (1991) [Infranuclear neurogenic bladder secondary to primary extradural hydatidosis]. Arch Esp Urol 44: 1198-1200.

102. Volobuev Iu M, Volobueva A (1991) [Experience with the treatment of brain and spinal cord echinococcosis]. Klin Khir: 53.

103. Casillo A, Bonetti MG, Scarabino T, Armillotta M (1992) [Spinal localization of hydatid cysts. Study with magnetic resonance]. Radiol Med 83: 297-299.

104. Hernigou P, Nabih A, Goutallier D (1992) [Vertebral hydatidosis. Complications, contribution of modern imaging]. Rev Rhum Mal Osteoartic 59: 131-135.

105. Kunze V, Layer G, Bruning R, Nagele M (1992) ["Metastasizing" Echinococcus alveolar of the liver]. Radiologe 32: 444-447.

106. Ogut AG, Kanberoglu K, Altug A, Cokyuksel O (1992) CT and MRI in hydatid disease of cervical vertebrae. Neuroradiology 34: 430-432.

107. Cancrini A, Jr., Bellotti C, Santoro A, Quagliarini L, Tossini A, et al. (1993) [Anterior approaches to the spinal column: considerations of the surgical technic]. G Chir 14: 92-98.

108. Guo HR, Lu YJ, Bao YH, Zhang TR (1993) Parasellar epidural hydatid cysts. Neurosurgery 32: 662-665.

109. Dahlbergh JO, Serrander R, Lindstam H (1994) [A case study of spinal echinococcosis. Effective management with chemotherapy plus surgery]. Lakartidningen 91: 2612-2614.

110. Ozek MM (1994) Complications of central nervous system hydatid disease. Pediatr Neurosurg 20: 84-91.

111. Querol JM, Perez M, Granda D, Cunat A (1994) [Atlanto-axial luxation secondary to cervical vertebral abscess]. Enferm Infecc Microbiol Clin 12: 511-513.

112. Druschky KF, Niederstadt T, Jourdan W, Stoltze D, Heckl R (1995) [High-grade transverse syndrome caused by echinococcus cysts]. Nervenarzt 66: 136-139.

113. Ousehal A, Adil A, El Azhari A, Kadiri R (1995) [Spinal cord compression disclosing rib hydatidosis]. J Radiol 76: 1093-1095.

114. Tikhodeev SA, Sovetova NA, Miroliubov SN (1995) [Spinal echinococcosis]. Vestn Rentgenol Radiol: 53-56.

115. Bulleri A, Tozzini A, Bianchi MC, Orsitto E, Pieri L (1996) [Vertebral echinococcosis. A case studied with computerized tomography and magnetic resonance]. Radiol Med 92: 130-132.

116. Sami A, Elazhari A, Ouboukhlik A, Elkamar A, Jiddane M, et al. (1996) [Hydatid cyst of the spine and spinal cord. Study of 24 cases]. Neurochirurgie 42: 281-287.

117. Tedeschi E, Camera L, Bartolomeo De Iuri A, Palescandolo P, Belfiore G, et al. (1996) [Spinal cord compression in systemic hydatidosis: study of a case with magnetic resonance and computerized tomography]. Radiol Med 92: 315-317.

118. Tsitouridis I, Dimitriadis AS (1997) CT and MRI in vertebral hydatid disease. Eur Radiol 7: 1207-1210.

119. Turgut M (1997) Hydatid disease of the spine: a survey study from Turkey. Infection 25: 221-226.

120. Berk C, Ciftci E, Erdogan A (1998) MRI in primary intraspinal extradural hydatid disease: case report. Neuroradiology 40: 390-392.

121. Chang KH, Han MH (1998) MRI of CNS parasitic diseases. J Magn Reson Imaging 8: 297-307.

122. Mazyad MA, Mostafa MM, Morsy TA (1998) Spinal cord hydatid cysts in Egypt. J Egypt Soc Parasitol 28: 655-658.

123. Normelli HC, Aaro SI, Follin PH (1998) Vertebral hydatid cyst infection (Echinococcus granulosus): a case report. Eur Spine J 7: 158-161.

124. Pneumatikos J, Frangides C, Malizos K, Tsagourias M, Nakos G (1998) Acute myelinolysis in the cervical spinal cord. J Trauma 44: 562-564.

125. Prousalidis J, Tzardinoglou K, Sgouradis L, Katsohis C, Aletras H (1998) Uncommon sites of hydatid disease. World J Surg 22: 17-22.

126. Tuzun M, Hekimoglu B (1998) Hydatid disease of the CNS: imaging features. AJR Am J Roentgenol 171: 1497-1500.

127. Bruschi F, Ortona E, Ioppolo S, Siracusano A, Bonadio M (1999) Immunochemical and molecular characterization of vertebral hydatid fluid. Scand J Infect Dis 31: 322-323.

128. Janni A, Davini F, Lucchi M, Consoli V, Angeletti CA (1999) Combined approach to intrathoracic lesions invading the spinal cord. J Cardiovasc Surg (Torino) 40: 897-899.

129. Mazyad MA, Morsy TA, Habib KS (1999) Vertebral unilocular hydatidosis in a shepherd and his wife. J Egypt Soc Parasitol 29: 547-550.

130. Savas R, Calli C, Alper H, Yunten N, Ustun EE, et al. (1999) Spinal cord compression due to costal Echinococcus multilocularis. Comput Med Imaging Graph 23: 85-88.

131. Zahawi HM, Hameed OK, Abalkhail AA (1999) The possible role of the age of the human host in determining the localization of hydatid cysts. Ann Trop Med Parasitol 93: 621-627.

132. Altinors N, Bavbek M, Caner HH, Erdogan B (2000) Central nervous system hydatidosis in Turkey: a cooperative study and literature survey analysis of 458 cases. J Neurosurg 93: 1-8.

133. Berk C, Ciftci E (2000) Hydatid disease. J Neurosurg 93: 181-182.

134. Dobato JL, Barriga FJ, Pareja JA, Vela L (2000) [Extrapontine myelinolyses caused by iatrogenic hypernatremia following rupture of a hydatid cyst of the liver with an amnesic syndrome as sequela]. Rev Neurol 31: 1033-1035.

135. Salaverria I, Ortuno F, del Rio F, Prado LM (2000) [Anaphylactic reaction and epigastralgia in a patient with an antecedent of pulmonary hydatidosis. Enferm Infecc Microbiol Clin 18: 359-360.

136. Danila N, Chifan M, Prescornita L, Andronic D, Tarasi C, et al. (2001) [Rare location of a hydatid cyst- in the upper mediastinum migrating into the spinal channel]. Rev Med Chir Soc Med Nat Iasi 105: 573-575.

137. Diedrich O, Kraft CN, Zhou H, Sommer T, Perlick L, et al. (2001) [Orthopedic aspects of osseous echinococcosis--radiologic diagnosis, current surgery and drug therapy aspects]. Z Orthop Ihre Grenzgeb 139: 261-266.

138. El Andaloussi M, Yousri B, Aboumaarouf M (2001) [Vertebral hydatidosis: three case reports]. Rev Chir Orthop Reparatrice Appar Mot 87: 392-396.

139. Hassan FO, Shannak A (2001) Primary pelvic hydatid cyst: an unusual cause of sciatica and foot drop. Spine (Phila Pa 1976) 26: 230-232.

140. Kostas JP, Dailiana Z, Xenakis T, Beris AE, Kitsoulis P, et al. (2001) Back pain caused by benign tumors and tumor-like lesions of the thoracolumbar spine. Am J Orthop (Belle Mead NJ) 30: 50-56.

141. Nalbanski B, Popivanova P, Lachev V, Ivanov S, Tsekova K (2001) [A case of pregnancy with fetal malformation and an hepatic hydatid cyst]. Akush Ginekol (Sofiia) 42: 29-31.

142. Onal C, Unal F, Barlas O, Izgi N, Hepgul K, et al. (2001) Long-term follow-up and results of thirty pediatric intracranial hydatid cysts: half a century of experience in the Department of Neurosurgery of the School of Medicine at the University of Istanbul (1952-2001). Pediatr Neurosurg 35: 72-81.

143. Solli P, Carbognani P, Cattelani L, Baldi P, Rusca M (2001) Unusually located hydatid cysts miming a pulmonary tumor invaliding the spine. J Cardiovasc Surg (Torino) 42: 147-149.

144. Stabler A, Reiser MF (2001) Imaging of spinal infection. Radiol Clin North Am 39: 115-135.

145. Zlitni M, Ezzaouia K, Lebib H, Karray M, Kooli M, et al. (2001) Hydatid cyst of bone: diagnosis and treatment. World J Surg 25: 75-82.

146. Hughes AJ, Biggs BA (2002) Parasitic worms of the central nervous system: an Australian perspective. Intern Med J 32: 541-553.

147. Natarajan S, Manoli S, Basnyat PS, Bransom CJ, Jones RD (2002) Spontaneous rupture of primary musculoskeletal hydatid cyst. Surgery 132: 533-534.

148. Pamir MN, Ozduman K, Elmaci I (2002) Spinal hydatid disease. Spinal Cord 40: 153-160.

149. Turgut AT, Turgut M (2002) Re: Vertebral hydatid cyst infection (Echinococcus granulosus): a case report. Eur Spine J 11: 393-394; author reply 395.

150. Kilic D, Erdogan B, Habesoglu MA, Hatipoglu A (2003) Multiple primary chest wall hydatid cysts associated with spinal canal involvement. Interact Cardiovasc Thorac Surg 2: 395-397.

151. Baleriaux DL, Neugroschl C (2004) Spinal and spinal cord infection. Eur Radiol 14 Suppl 3: E72-83.

152. Mellado JM, Perez del Palomar L, Camins A, Salvado E, Ramos A, et al. (2004) MR imaging of spinal infection: atypical features, interpretive pitfalls and potential mimickers. Eur Radiol 14: 1980-1989.

153. El-Arousy MH, Ismail SA (2005) Cerebrospinal echinococcosis: serodiagnosis using different hydatid cyst fluid antigens. J Egypt Soc Parasitol 35: 193-204.

154. Kadioglu HH, Malcok UA, Senguli G, Aydin IH (2005) Alveolar hydatid disease of the spine causing paraplegia. Neurosciences (Riyadh) 10: 180-182.

155. Ramos Fernandez R, Navia Roque J, Garcia Sabrido JL, Rodriguez Rodrigalvarez R, Valdecantos Montes E, et al. (2005) [Anterior surgical approach to T12 and L1 in recurring vertebral hydatidosis: anesthetic management]. Rev Esp Anestesiol Reanim 52: 545-549.

156. Rebai R, Kacem AH, Gdoura F, Chabchoub I, Ben Mansoor H (2005) Where an unusual lesion is treated aggressively. Spine J 5: 579-580.

157. Khazim RM (2006) Spinal hydatid disease. South Med J 99: 114.

158. Nurkalem Z, Atmaca H, Kayacioglu I, Uslu N, Gorgulu S, et al. (2006) Hydatid disease involving the left ventricle: a case of unusual combination. Int J Cardiol 112: e30-32.

159. Braham E, Ayadi-Kaddour A, Abid L, Ismail O, Smati B, et al. (2007) [Primary hydatidosis of the chest wall. Report of 5 cases]. Tunis Med 85: 704-708.

160. Fernandez-Esparrach G, Gimeno-Garcia AZ, Ayuso JR, Gines A, Bordas JM (2007) Vertebral prosthetic arthrodesis migration to the esophageal lumen: a rare cause of dysphagia. Endoscopy 39 Suppl 1: E188.

161. Makni F, Hachicha L, Mseddi F, Hammami H, Cheikhrouhou F, et al. (2007) [Contribution of Western blotting to the diagnosis of hydatidosis]. Bull Soc Pathol Exot 100: 171-173.

162. Sanal HT, Kocaoglu M, Bulakbasi N, Yildirim D (2007) Pelvic hydatid disease: CT and MRI findings causing sciatica. Korean J Radiol 8: 548-551.

163. Zidane A, Arsalane A, Atoini F, El Mostarchid B, Kabiri EH (2007) [Multifocal (spinal cord, thorax, abdomen) severe complicated hydatid disease]. Rev Pneumol Clin 63: 62-64.

164. Izci Y, Tuzun Y, Secer HI, Gonul E (2008) Cerebral hydatid cysts: technique and pitfalls of surgical management. Neurosurg Focus 24: E15.

165. Kalaci A, Sevinc TT, Yanat AN (2008) Sciatica of nondisc origin: hydatid cyst of the sciatic nerve. Case report. J Neurosurg Spine 8: 394-397.

166. Nieto JM, Vives I, Jimenez JA, Gonzalez MA, Guerrero E, et al. (2008) [Anesthetic management of sacroiliac-vertebral echinococcosis]. Rev Esp Anestesiol Reanim 55: 434-437.

167. Rodallec MH, Feydy A, Larousserie F, Anract P, Campagna R, et al. (2008) Diagnostic imaging of solitary tumors of the spine: what to do and say. Radiographics 28: 1019-1041.

168. Zani A, Cozzi DA (2008) Giovanni Battista Morgagni and his contribution to pediatric surgery. J Pediatr Surg 43: 729-733.

169. Dagtekin A, Koseoglu A, Kara E, Karabag H, Avci E, et al. (2009) Unusual location of hydatid cysts in pediatric patients. Pediatr Neurosurg 45: 379-383.

170. Turgut AT, Turgut M (2009) Intradural extramedullary primary hydatid cyst of the spine in a child: a very rare presentation. Eur Spine J 18: 1234-1235; author reply 1236.

171. Elmaataoui A, Miss E, Esselmani H, Derfoufi O, Sabri M, et al. (2010) [Hydatid disease of the spine: a case report]. Ann Biol Clin (Paris) 68: 729-732.

172. Moraux A, Kermarrec E, Czarnecki E, Boutry N, Demondion X, et al. (2010) [Spinal infections: typical and atypical imaging features]. J Radiol 91: 1049-1056.

173. Nourbakhsh A, Vannemreddy P, Minagar A, Toledo EG, Palacios E, et al. (2010) Hydatid disease of the central nervous system: a review of literature with an emphasis on Latin American countries. Neurol Res 32: 245-251.

174. Rando K, Harguindeguy M, Leites A, Ettlin A, Gonzalez S, et al. (2010) [Quality standards in liver surgery: influence of multidisciplinary team work and patient centralization]. Acta Gastroenterol Latinoam 40: 10-21.

175. Turgut M, Turgut AT (2010) Spinal intradural extramedullary hydatidosis: report of 3 cases. Neurosurgery 67: E1471-1472.

176. Diktas H, Cakmak S, Turhan V, Kantemir A, Gulec B, et al. (2011) [Hydatid cyst presenting with multiple recurrence and pelvic involvement: case report]. Turkiye Parazitol Derg 35: 178-180.

177. Ouadnouni Y, Bouchikh M, Achir A, Smahi M, Msougar Y, et al. (2011) [Hydatid disease of the ribs]. Rev Mal Respir 28: 306-311.

178. Venara A, Mehinto DK, Lermite E, Chabasse D, Hamy A, et al. (2011) [Rare primary localization of hydatidosis]. Presse Med 40: 438-442.
